# Drilling Strategies to Improve the Geometrical and Dimensional Accuracy of Deep through Holes Made in PA6 Alloy

**DOI:** 10.3390/ma16010110

**Published:** 2022-12-22

**Authors:** Mateusz Bronis, Edward Miko, Krzysztof Nozdrzykowski

**Affiliations:** 1 Department of Machine Design and Machining, Kielce University of Technology, al. Tysiaclecia Panstwa Polskiego 7, 25-314 Kielce, Poland; 2 Department of Metrology and Unconventional Manufacturing, Kielce University of Technology, al. Tysiaclecia Panstwa Polskiego 7, 25-314 Kielce, Poland; 3 Faculty of Marine Engineering, Maritime University of Szczecin, ul. Wały Chrobrego 1-2, 70-500 Szczecin, Poland

**Keywords:** drilling, drilling strategy, hole quality, ANOVA, universal turning center

## Abstract

This article shows how different drilling strategies may affect the geometrical and dimensional accuracy of deep through holes. The tests were conducted on a three-axis direct-drive turning center. The holes were drilled in cylindrical PA6 aluminum alloy specimens 30 mm in length and 30 mm in diameter using 6 mm Ø VHM HPC TiAlN-coated twist drill bits. The cutting fluid was supplied to the cutting zone through the spindle. The experiments involved applying three strategies to drill deep through (5D) holes. The first required the workpiece to be fixed and the tool to perform both rotary and reciprocating motions. The second assumed that the workpiece performed the primary (rotary) motion whereas the tool moved in reciprocating motion. In the third strategy, the workpiece and the tool rotated in opposite directions and the tool also performed a reciprocating motion. The straightness, roundness, cylindricity, and diameter errors were the key output parameters in the analysis of the geometrical and dimensional accuracy of holes. The Taguchi orthogonal array design of experiment (DOE) was employed to determine the effects of the input (cutting) parameters (i.e., spindle speed and feed per revolution) and the type of hole making strategy on the hole errors by means of multi-factor statistical analysis ANOVA. The use of the highest spindle speed (*n* = 4775 rpm), the highest feed per revolution ($f_{n}$ = 0.14 mm/rev) and strategy I resulted in the lowest values of the output parameters (STR = 22.7 µm, RON = 8.6 µm, CYL = 28.2 µm, and DE = 9.9 µm). Strategy I was reported to be the most effective for hole drilling in PA6 aluminum alloy because, irrespective of the values of the process parameters used, three out of four output parameters, i.e., straightness, roundness and diameter errors, reached the lowest values.

## 1. Introduction

Aluminum and aluminum alloys have been widely used in many industries and this is due to such properties as light weight, high resistance to corrosion, good electrical and thermal conductivity, and recyclability [1,2]. Still, the sales of these materials are predicted to rise from USD 147.2 bn (2018) to USD 189.8 bn by 2026 [3]. The most common applications of aluminum and aluminum alloys are in the automotive, construction, electrical engineering and electronics, shipbuilding, and obviously aviation industries [1]. As indicated in [4], aluminum is the key material (80% of all materials) used in Boeings 747, 757, 767, and 777.

Unlike non-ferrous metals or their alloys, aluminum alloys are relatively easy to machine. However, hole making may present a challenge mainly because aluminum alloys have high ductility; this property may be responsible for the formation of long ductile chips, which are difficult to remove from the cutting zone [5]. Recently, investigations into the machining of aluminum alloys have concentrated on deep hole making where high geometrical and dimensional accuracy is achieved.

From the review of the literature, it is clear that there have been many studies on the relationship between the cutting parameters and the hole-making accuracy for aluminum alloys. Szwajka and Zielińska-Szwajka [6], for example, dealt with hole roundness and diameter errors as a linear function of the feed rate for constant values of the cutting speed (7, 24, or 28 m/min). The results show that the most accurate hole was obtained at *v_c_* = 7 m/min and the highest feed rate. Giasin et al. [7,8] analyzed roundness and diameter errors, measured at the hole entrance and exit, in relation to two process parameters: the spindle speed (*n* = 1000, 3000, 6000, and 9000 rpm) and the feed rate (*f* = 100, 300, 600, and 900 mm/min). They found that the roundness error increased at *n* = 3000 or 6000 rpm, and decreased at *n* = 1000 or 9000 rpm. Angelone et al. [9] investigated how one constant value of the feed per revolution and two values of the spindle speed (*n* = 3000 or 4500 rpm) affected the hole drilling accuracy and roundness error. They reported the highest accuracy and the smallest error of holes at *n* = 4500 rpm and $f_{n}$ = 0.15 mm/rev. Gowda et al. [10,11] considered the impact of the process parameters (*n* = 360, 490, 680 rpm, $f_{n}$ = 0.095, 0.19, 0.285 mm/rev), the drill bit diameter (d = 6, 8, and 10 mm), and the drilling time (t = 30, 60, and 90 sec) on the hole roundness and cylindricity. They show that the use of the lowest spindle speed of 360 rpm and the lowest feed per revolution of 0.095 mm/rev resulted in the lowest roundness error; however, the same spindle speed and $f_{n}$ = 0.285 mm/rev gave the lowest cylindricity error. Prakash et al. [12] proposed a mathematical model to predict the relationship between the hole accuracy and the type (M2 HSS, M35 HSS) and diameter (d = 4, 8, and 12mm) of the drill bit. They studied the influence of three different values of each process parameter (*n* = 80, 160, and 244 rpm, $f_{n}$ = 0.1, 0.125, 0.15 mm/rev). Çiçek, Kivak, and Ekici [13] analyzed the influence of the process parameters (*v_c_* = 12, 14, and 16 m/min, $f_{n}$ = 0.04, 0.06, and 0.08 mm/rev) and the drill bit categories (conventionally heat treated—CHT, cryogenically treated, CT—cryotempered—CTT) on the hole roundness. The coefficient of determination obtained for the hole roundness error reached 0.91. They concluded that the hole roundness was dependent mainly on the feed per revolution and the cutting speed (64.365%). The best results were obtained for the cryotempered drill at *v_c_* = 12 m/min and $f_{n}$ = 0.06 mm/rev. The research described in [14,15] focused on the influence of the drill bit coating on the hole straightness, roundness and diameter errors, measured as a function of the number of drill bits used. Four types of drill bits were considered: uncoated HSS, HSS+TiAlN, HSS+TiN, and HSS+%5Co. The HSS+%5Co tool was reported to reduce the hole roundness and diameter errors considerably. Sandeep, Ajay, and Jagadesh [16] studied the influence of the spindle speed (*n* = 400, 800, 1200, 1600, 2000, and 2300 rpm) and the type of cutting fluid (none present, blasocut, graphite, and molybdenum disulfide) on the hole roundness error. The most accurate holes were made when the cutting was performed with no coolant or in the presence of blasocut. The effects of the kinematic systems and the process parameters on the dimensional and geometrical accuracy of holes were discussed in [17,18,19]. From the experimental data, it was clear that the kinematic system had a significant impact only on some of the output parameters related to the geometrical and dimensional hole accuracy. Yoon Par et al. [20] investigated how the process parameters, i.e., the spindle speed (*n* = 600, 1800, or 3000 rpm), the feed per revolution ($f_{n}$ = 0.04, 0.12, and 0.2 mm/rev) and the type of drill bit (HSS; sintered carbide) affected the hole roundness and the hole accuracy. Çiçek and Uçak [21] made a step forward; they extended their research by adding another factor, i.e., the type of cooling (dry, LN_2_, and cryogenic conditions). The presence of LN_2_ improved the hole roundness from 20% to 70%, depending on the values of the process parameters. Kurt et al. [22] proposed a model to predict the hole diameter accuracy relative to the drilling depth (d = 15; 25 mm), the drill bit coating (HSS+TiN, HSS+TiAlN, or uncoated HSS), the cutting speed (*v_c_* = 30, 45, and 60 m/min), and the feed per revolution ($f_{n}$ = 0.15, 0.2, or 0.25 mm/rev). There was a good fit (88%) of the predicted data to the measured data. A completely different approach was proposed in [23], where the study involved developing mathematical models to determine if the spindle speed (*n* = 2000, 3500, and 5000 rpm), the feed rate (*f* = 5, 10, and 15 mm/min), and the drill bit pressure (P = 2, 4, and 6 bars) on the hole cylindricity and diameter errors. It was found that the most important input parameter was the spindle speed (42% for DE and 50.48% for RON). Khanna et al. [24] investigated how the type of cooling (dry or cryogenic conditions) affected the hole quality (cylindricity and roundness errors). They used a constant value of the cutting speed *v_c_* = 19 m/min and a constant value of the feed per revolution $f_{n}$ = 0.02 mm/rev. They indicated that cryogenic cooling reduced the cylindricity and roundness errors by 77% and 51%, respectively. Aized and Amjad [25] formulated models for determining the hole diameter, roundness, and cylindricity errors in relation to the spindle speed (*n* = 100, 200, 400, 600, 800, and 1000 rpm), feed rate (*f* = 5, 10, 15, 20, and 25 mm/min), and the drilling method (single and multiple pass penetration). The relationship between the spindle speed and the hole cylindricity and diameter errors was discussed in [26]. Singh, Kumar and Saini [27] analyzed the impact of the spindle speed (*n* = 800, 1200, and 1600 rpm), feed per revolution ($f_{n}$ = 0.1, 0.14, and 0.18 mm/rev), and the drill tip point angle (118, 127, and 135 degrees) on the hole diameter error. The research data indicated that the hole making accuracy was dependent mainly on the point angle.

From the review of the literature, it is evident that there have been hardly any studies dealing with the effects of the drilling strategy on the accuracy of holes in PA6 aluminum alloy. The novelty of the research presented in this article is the use of statistical analysis to compare the effects of three drilling strategies. The hole-making strategy and the process parameters will be considered as the primary factors contributing to the dimensional and geometrical accuracy of holes (straightness, roundness, cylindricity, and diameter errors) drilled in PA6 aluminum alloy. The study involved employing mathematical models to predict the errors in relation to the input parameters. The most suitable input parameters were selected to obtain the desirable values of the output parameters.

## 2. Materials and Methods

The primary goal of this study was to compare three through hole drilling strategies in order to determine which provided the highest dimensional and geometrical accuracy. The four output parameters used in this analysis were the hole straightness, roundness, cylindricity, and diameter errors. The experiments also aimed to assess how the drilling parameters, i.e., the spindle speed (*n*) and the feed per revolution ($f_{n}$) affected the hole accuracy. The holes were drilled using a Gildemeister CTX ALPHA 500 (DMG Mori, Bielefeld, Germany) turning center equipped with a 12-position turret (VDI30 DIN 5480 direct drive, Sauter, Metzingen, Germany). The holes were made using a 6 mm Ø VHM HPC twist drill bit coated with titanium aluminum nitride (TiAlN). The cylindrical specimens were 30 mm in length and 30 mm in diameter. The drill bit was mounted in an axially driven holder (VDI30 SAUTER 113180, Sauter, Metzingen, Germany) using an ER25 DIN 5480 collet chuck (Orion, Ludwigsburg, Germany). In the experiments, the cutting fluid was supplied to the cutting zone through the spindle, although the holding system also allowed for internal cooling through the tool and the external nozzle.

The material used for the experiments was PA6 3.1325 aluminum alloy, characterized by high ductility, formability, and machinability, but low corrosion resistance and weldability. The PA6 alloy is suitable for engineering structures as well as machine and automotive components operating under low and elevated temperature conditions. Table 1 shows the chemical composition determined by means of a Phenom XL scanning electron microscope (Thermo Fisher Scientific, Grand Island, NE, USA).

**Figure 1:**
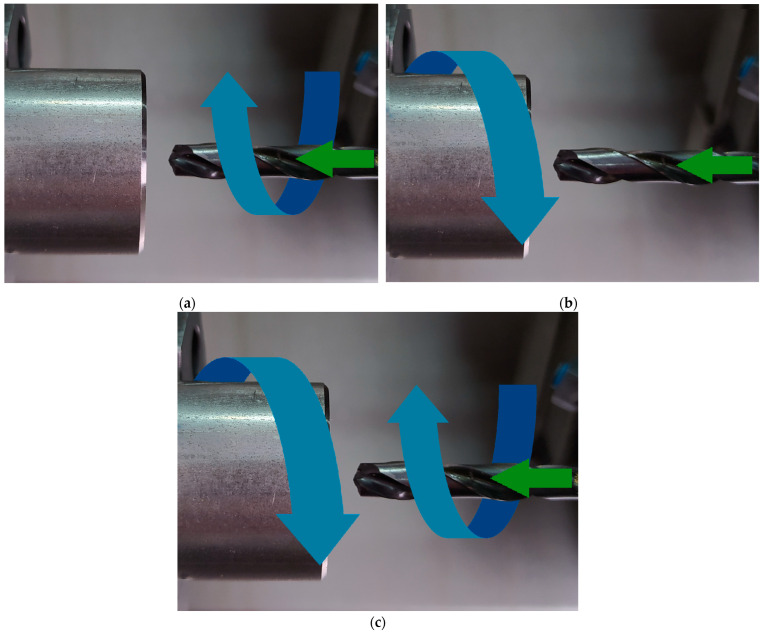
Drilling strategies: (**a**) Strategy I, (**b**) Strategy II, and (**c**) Strategy III.

Figure 1 illustrates three different strategies of drilling in the material tested. Figure 1a shows the first strategy, where the workpiece is fixed while the tool performs both the primary rotary motion and the secondary reciprocating motion. Figure 1b depicts the second strategy, where the workpiece performs the primary rotary motion and the tool moves along a straight line, parallel to the axis of rotation of the workpiece. Figure 1c shows the third strategy, where the workpiece and the tool rotate in opposite directions and the tool also performs a reciprocating motion.

Equation (1), used to analyze the deep through hole drilling strategies, is based on the difference between the tool speed and the spindle speed.

Strategy = *n_n_* − *n*, (1)

where: Strategy—strategy, *n_n_*—tool speed, and *n*—spindle speed.

The experiments involved testing 27 specimens using three different values of the spindle speed (*n* = 3183, 3979, and 4775 rpm), three different values of the feed per revolution ($f_{n}$ = 0.1, 0.12, and 0.14 mm/rev), and three different drilling strategies (I, II, and III). The Taguchi orthogonal array (3 × 3 × 3) was applied. The test conditions are shown in Table 2.

**Figure 2:**
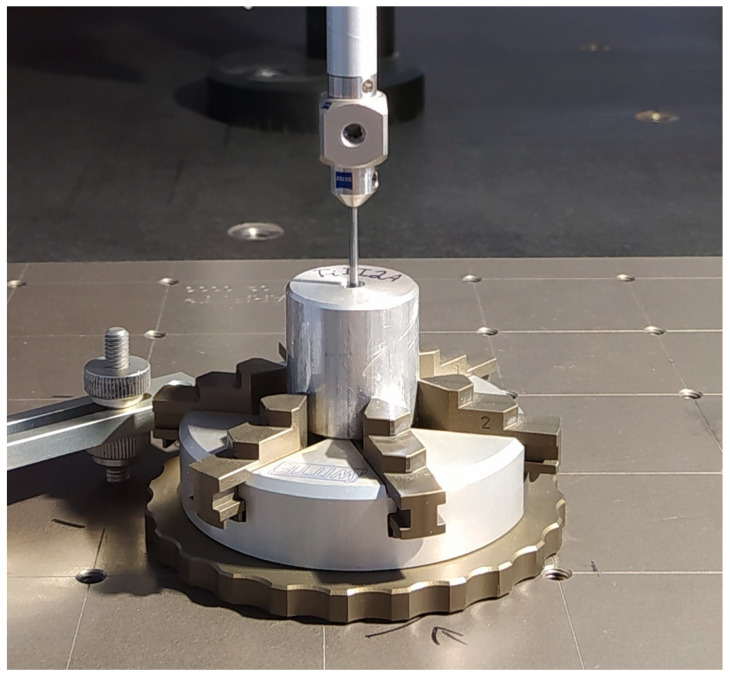
Measurement of a specimen mounted on the coordinate measuring machine.

**Figure 3:**
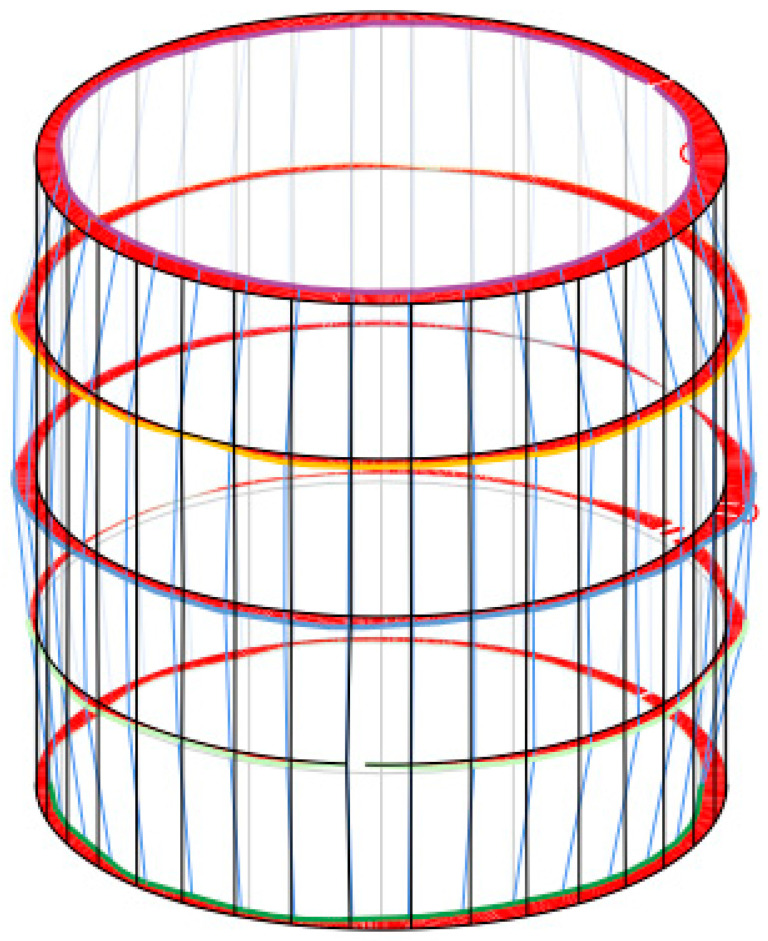
Optical data generated by the coordinate measuring machine for the hole drilled in Experiment No. 6.

Figure 2 depicts how the specimens were measured using a Zeiss Prismo Navigator coordinate measuring machine (Zeiss, Oberkochen, Germany, MPE_E 0.9 + L/350 µm, MPE_P 1.0 µm, MPE_RON_t_ = 1 µm, MPE_THP = 1.9 µm). Figure 3 depicts optical data generated by the coordinate measuring machine. The data were collected by a 1.5 mm Ø ruby tip probe travelling with a speed of 5 mm/s. The straightness error was determined by measuring how four generating lines shifted (relative to one another) by 90°. The diameter and roundness errors were obtained by measuring five circles shifted (relative to one another) by 7.5 mm. The cylindricity error was established by finding the maximum inscribed cylinder, which required measuring roundness profiles of a hole in five cross-sections.

## 3. Results

### 3.1. Metrological Analysis

As indicated in Section 2 of this article, the geometry of the cylindrical holes made using different drilling strategies was assessed by measuring the straightness of the generating lines and the roundness in five cross-sections. The measurement results were used to determine the error of straightness of the generating lines and the roundness error for each cross-section. The roundness data were also employed to calculate the cylindricity and diameter errors. The cylindricity error is the greatest contributor to the geometrical and dimensional accuracy of holes. Since the cylindricity error is constituted by the relative change in the hole diameter, the roundness error obtained for each cross-section and the concentricity error in relation to the nominal axis of the hole, the use of the measuring strategy based on roundness data is the most favorable approach used to assess the hole geometry. The cylinder error was estimated with reference to the nominal cylinder determined using the least squares method, which is now considered the most suitable for this purpose.

**Figure 4:**
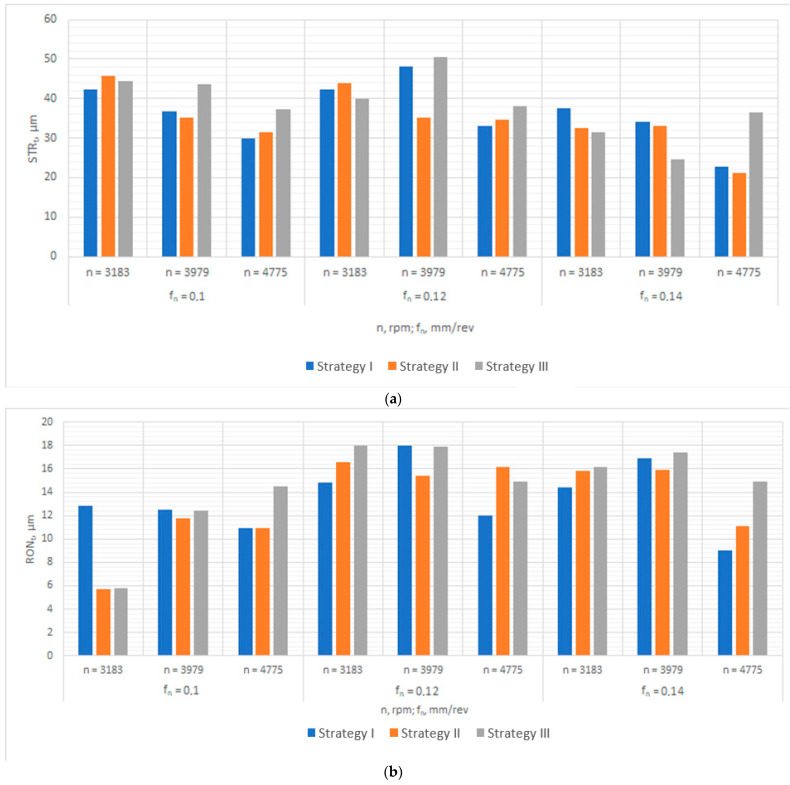

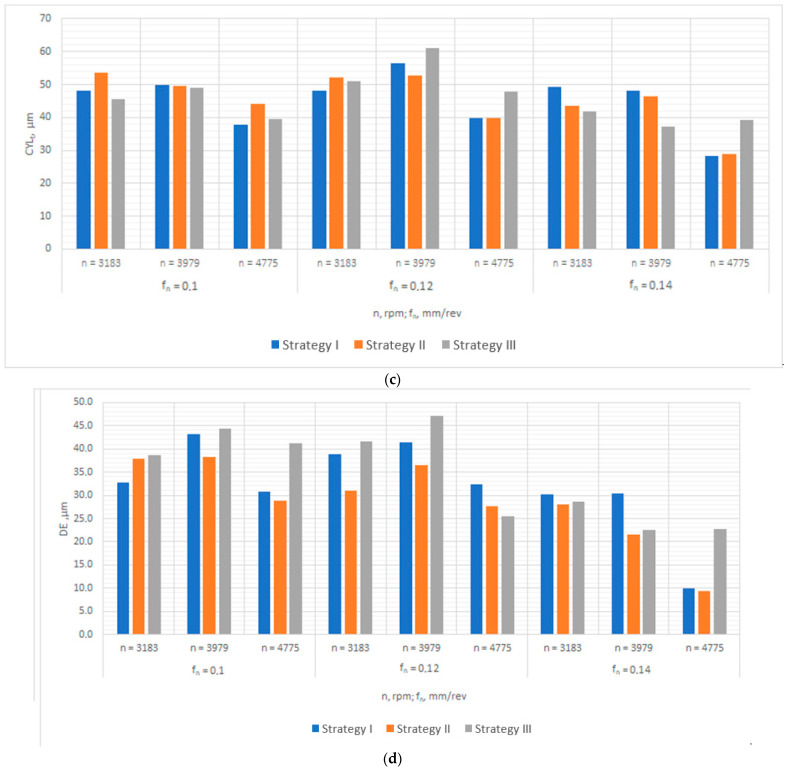
Graphical representation of the measured data: (**a**) straightness error, (**b**) roundness error, (**c**) cylindricity error, (**d**) diameter error.

**Figure 5:**
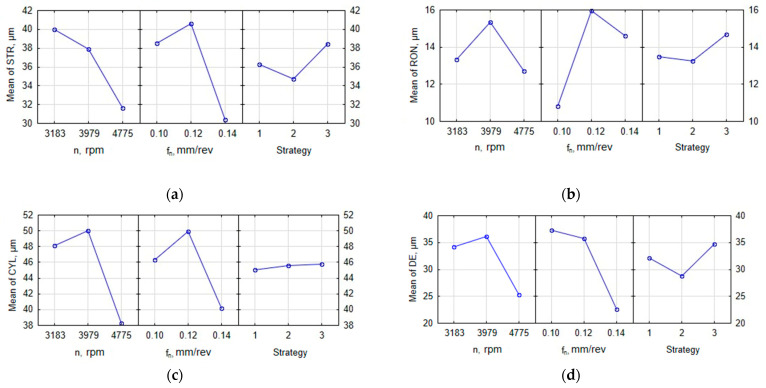
Main effects plots: (**a**) straightness error, (**b**) roundness error, (**c**) cylindricity error, and (**d**) diameter error.

As the data sets obtained from the experiments were large, the data were represented graphically (Figure 4 and Figure 5) so that they would be easier to read, analyze, and interpret.

As can be seen from Figure 4a, the hole straightness error, determined by measuring the straightness of the generating lines of the cylindrical hole, are dependent on the process parameters and the hole drilling strategy. The lowest values were registered in experiments No 1 and 2, carried out at *n* = 4775 rpm and $f_{n}$ = 0.14 mm/rev using strategies I and II, respectively. The straightness error reached 22.7 µm and 21.1 µm, respectively. The highest straightness error of 50.5 µm was obtained in experiment No 15, performed at *n* = 3979 rpm and $f_{n}$ = 0.12 mm/rev using strategy III.

Figure 4b shows how the hole roundness error is affected by the input parameters and the drilling strategy. The analysis indicates that the roundness error was the lowest in experiments 26 and 27; the error reached 5.7 µm and 5.8 µm, respectively. These experiments were conducted at *n* = 3183 rpm and $f_{n}$ = 0.1 mm/rev, using strategies II and III, respectively. The use of strategy I and the same process parameters (*n* = 3183 rpm and $f_{n}$ = 0.1 mm/rev) resulted in an approximately twice as high roundness error of 12.8 μm.

Figure 4c depicts the relationship between the cylindricity error and the hole drilling conditions. The analysis reveals that in experiments Nos. 1 and 2, carried out at *n* = 4775 rev/min and $f_{n}$ = 0.14 mm/rev, the values of the cylindricity error were the lowest, reaching 28.2 μm and 28.8 μm, respectively. Experiment No. 1 was performed using drilling strategy I, while in experiment No. 2, strategy II was employed. The highest value of the cylindricity error, i.e., 61.2 µm, was reported in experiment No. 15, at *n* = 3979 rpm and $f_{n}$ = 0.12 mm/rev, when strategy III was used.

From Figure 4d illustrating the diameter error against the input parameters and the drilling strategy, it is apparent that the lowest values of the hole diameter error (9.9 μm and 9.3 µm) were obtained at *n* = 4775 rev/min, $f_{n}$ = 0.14 mm/rev and drilling strategies I and II, respectively, which were the same conditions as those in which the lowest straightness and cylindricity errors were reported. When the hole making was performed at the same values of the process parameters but strategy III was employed, the hole diameter error was twice as high (22.8 µm).

The experimental results suggest that when through holes were drilled at the highest values of the input parameters (*n* = 4775 rpm and $f_{n}$ = 0.14 mm/rev) using strategy I, all the output parameters were the lowest.

The main effect plots in Figure 5 illustrate how each output parameter is dependent on the spindle speed, feed per revolution and drilling strategy.

The analysis of the plots in Figure 5a, showing the straightness error against the input parameters *n* and *f* and the drilling strategy, reveals that at the highest spindle speed, the average straightness error of the generating lines of the cylindrical hole reached 31.8 µm. The use of the highest feed per revolution resulted in the lowest straightness error of 30.3 µm. The straightness error reported for strategy II was slightly higher, reaching 34.6 µm.

As can be seen from Figure 5b depicting the roundness error against the input parameters *n* and *f* and the drilling strategy, the highest spindle speed led to the lowest average hole roundness error (RON_avg_ = 12.7 µm). The most appropriate value of the feed required to minimize the hole roundness error was 0.1 mm/rev (RON_avg_ = 10.8 µm). The most effective strategies were strategies I and II. For both strategies, similar values of the hole roundness error were obtained (13.5 and 13.3 µm, respectively). Strategy III was reported to be the least effective in the minimization of the hole roundness error (RON_avg_ = 14.6 µm).

From the diagrams in Figure 5c, illustrating the relationship between the cylindricity error and the input parameters *n* and *f* as well as the drilling strategy, it is clear that the use of the highest value of the spindle speed (4775 rev/min) resulted in an average cylindricity error of 38.1 µm. When the highest feed per revolution of 0.14 mm/rev was used, the cylindricity error reached 40.2 µm. The effect of the drilling strategy on the cylindricity error was negligible; the difference between the highest and lowest values was only 1.3 µm. The cylindricity error was reported to be the lowest when strategy I or II was used.

The plots in Figure 5d, depicting the changes in the diameter error depending on the input parameters *n* and *f* and the drilling strategy suggest that the most accurate hole was obtained at *n* = 4775 rev/min (DE_avg_ = 25.3 µm). An increase in the feed per revolution led to an increase in the dimensional accuracy of the hole. Strategy II was reported to be the best (DE_avg_ = 28.7 µm) and strategy III the least favorable (DE_avg_ = 34.7 µm).

From the experimental data, it can thus be concluded that the highest geometrical and dimensional accuracy of holes drilled in PA6 alloy was obtained for strategy II. Three out of four output parameters had the lowest values when this strategy was used. Similar observations were made for the highest spindle speed (*n* = 4775 rev/min). The findings for the cylindricity error confirm this observation because this universal parameter is the most suitable to assess the hole geometry.

### 3.2. Statistical Analysis (ANOVA and GRA)

The measurement results were analyzed using the analysis of variance (ANOVA). This statistical method helped determine the effects of each input parameter on each output parameter. As 27 different combinations of the input parameters were studied, the Taguchi L27 array was applied. All the calculations were performed using Statistica software at a 95% confidence interval and a 5% significance level. The results of the statistical analysis are given in Table 3 and Table 4.

The estimated values of SS and MS were used to mathematically determine the F value. The significance of each analysis was established on the basis of the F value. From Table 3 and Table 4, it can be concluded that the model assumes *p* values to be much smaller than 0.05, which confirms the significance of the analyses. Table 3 shows that for the straightness error model (Equation (2)), the coefficient of determination reached 72.13%, indicating a good fit. The straightness error was found to be dependent on the feed per revolution (73.55%) and drilling strategy (19.87%). For the hole roundness error (Equation (3)), the coefficient of determination was 81.10%, which ensured a good fit, as was the case with the cylindricity error model. The input parameters responsible for the roundness error were the feed per revolution (54.03%) and the spindle speed (34.42%). From Table 4, it is evident that for the cylindricity error (Equation (4)), the coefficient of determination was 78.88%, which also showed a very good fit. The feed per revolution (49.09%) and the spindle speed (47.64%) were the key input parameters. For the hole diameter error (Equation (5)), the coefficient of determination reached 87.63%, which indicates a very good fit. The input parameters that had the greatest influence were the spindle speed (49.43%) and feed per revolution (40.31%).

(2) $$STR=-155.12+1.25\cdot 10^{-2}\cdot n-3.07\cdot 10^{-6}\cdot n^{2}+3245,06\cdot f_{n}-15458,33\cdot f_{n}{}^{2}-4.1\cdot 10^{-4}\cdot Strategy-2.41\cdot 10^{-7}\cdot Strategy^{2}+6.59\cdot 10^{-2}\cdot n\cdot f_{n}-1.59\cdot 10^{-7}\cdot n\cdot Strategy+1.04\cdot 10^{-2}\cdot f_{n}\cdot Strategy$$

(3) $$RON=-226.37+4.35\cdot 10^{-2}\cdot n-3.61\cdot 10^{-6}\cdot n^{2}+2539.32\cdot f_{n}-8152.77\cdot f_{n}{}^{2}+2.35\cdot 10^{-3}\cdot Strategy-9.51\cdot 10^{-8}\cdot Strategy^{2}-1.22\cdot 10^{-1}\cdot n\cdot f_{n}-2.99\cdot 10^{-7}\cdot n\cdot Strategy-9.19\cdot 10^{-3}\cdot f_{n}\cdot Strategy$$

(4) $$CYL=-324.24+8.68\cdot 10^{-2}\cdot n-1.06\cdot 10^{-5}\cdot n^{2}+4088.96\cdot f_{n}-16625\cdot f_{n}{}^{2}-1.68\cdot 10^{-3}\cdot Strategy-9.17\cdot 10^{-8}\cdot Strategy^{2}-6.33\cdot 10^{-2}\cdot n\cdot f_{n}-1.17\cdot 10^{-7}\cdot n\cdot Strategy+1.75\cdot 10^{-2}\cdot f_{n}\cdot Strategy$$

(5) $$DE=-352.83+9.75\cdot 10^{-2}\cdot n-9.9\cdot 10^{-6}\cdot n^{2}+3864.25\cdot f_{n}-14472.22\cdot f_{n}{}^{2}-2.31\cdot 10^{-4}\cdot Strategy-2.62\cdot 10^{-7}\cdot Strategy^{2}-1.9\cdot 10^{-1}\cdot n\cdot f_{n}-8.59\cdot 10^{-8}\cdot n\cdot Strategy+8.44\cdot 10^{-3}\cdot f_{n}\cdot Strategy$$

**Figure 6:**
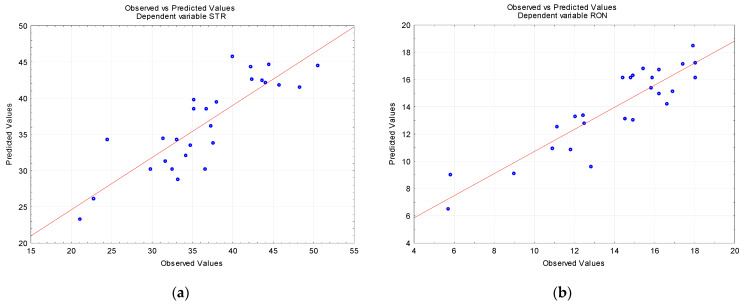

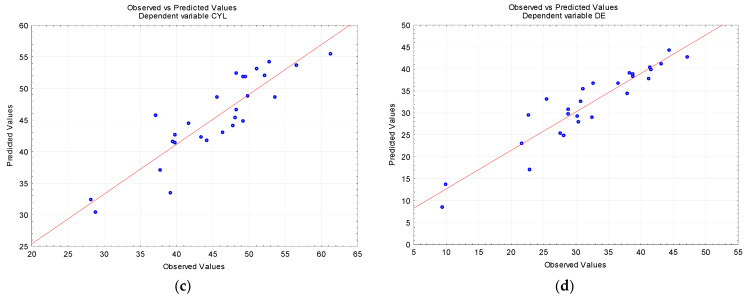
Observed vs. predicted values: (**a**) straightness error, (**b**) roundness error, (**c**) cylindricity error, (**d**) diameter error.

As can be seen from Figure 6, all the measured and predicted values indicate a good fit because for the straightness error (Figure 6a), the coefficient of determination, R^2^, was 0.7213, for the roundness error (Figure 6b) R^2^ = 0.8110, for the cylindricity error (Figure 6c) R^2^ = 0.7888, and for the diameter error (Figure 6d) R^2^ = 0.8763.

The input variables were optimized using the Grey relational analysis (GRA), which is a multi-objective optimization approach. In this case, the aim was to obtain the lowest possible value of each output parameter. Each output parameter (STR, RON, CYL, and DE) was given the same weight (all were equally significant). The analysis results are provided in Table 5. From the qualitative point of view (STR, RON, CYL, and DE), the most optimal process conditions were at *n* = 4775 rpm, *f_n_* = 0.14 mm/rev and Strategy I (experiment No. 1)

**Figure 7:**
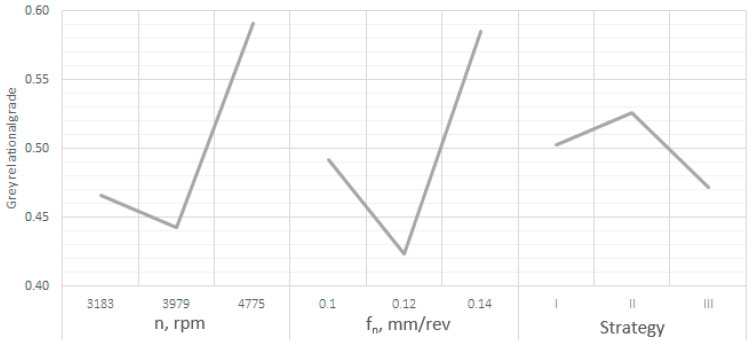
Response graph for mean Grey relational grade.

In order to isolate the effect of each input variable on the grey relational grade at different levels, a response graph for the grey relational grade was constructed, as shown in Figure 7. In general, the larger the gray relational degree, the better the performance characteristics [28,29]. It shows that the smallest average values of process output parameters will be obtained for *n* = 4775 rpm; *f_n_* = 0.14 mm/rev and Strategy II.

## 4. Conclusions

The purpose of the study described in this article was to determine how different drilling parameters and drilling strategies affected the geometrical and dimensional accuracy of deep through holes made in PA6 aluminum alloy. The experiments involved measuring the straightness, roundness, cylindricity, and diameter errors. The authors hope that the research results presented here will be of practical application to the manufacturing sector as they may be used to optimize the drilling process.

The following are the most important findings from the study:

- The Grey relational grade showed that for the highest spindle speed (*n* = 4775 rpm), the highest feed per revolution ($f_{n}$ = 0.14 mm/rev), and Strategy I, all the output parameters were the lowest (STR = 22.7 µm, RON = 8.6 µm, CYL = 28.2 µm, and DE = 9.9 µm).
- Strategy II was the best strategy to drill deep through holes in PA6 alloy as the lowest results were obtained for three out of four output parameters (straightness, roundness and diameter errors).
- The drilling strategy had a 20 percent contribution to the hole straightness error.
- The predicted data were similar to the measured data since high values of the coefficient of determination were observed (R^2^ = 0.7213 for STR, R^2^ = 0.811 for RON, R^2^ = 0.7888 for CYL, and R^2^ = 0.8763 for DE).
- The feed per revolution had a significant impact on the geometrical and dimensional accuracy of holes; the percent contributions were as follows: 74% for STR, 54% for RON, 49% for CYL, and 40% for DE.
- The statistical analysis ANOVA helped determine how each input parameter affected each output parameter.
- Future research on hole drilling will be extended to the measurement of burrs and surface roughness.

## Figures and Tables

**Table 1:** Composition of PA6 determined by SEM.

Alloying Elements (% Weight)							
Si	Fe	Cu	Mn	Mg	Cr	Zn	Ti
0.74	0.69	3.6	0.45	0.98	0.1	0.24	0.04

**Table 2:** Factors considered in the drilling experiments.

Experiment No.	*n*, rev/min	$f_{n},\;\mathrm{mm}/\mathrm{rev}$	Strategy No.	Strategy, Equation (1)
1	4775	0.14	1	4775
2	4775	0.14	2	−4775
3	4775	0.14	3	0
4	3979	0.14	1	3979
5	3979	0.14	2	−3979
6	3979	0.14	3	0
7	3183	0.14	1	3183
8	3183	0.14	2	−3183
9	3183	0.14	3	0
10	4775	0.12	1	4775
11	4775	0.12	2	−4775
12	4775	0.12	3	0
13	3979	0.12	1	3979
14	3979	0.12	2	−3979
15	3979	0.12	3	0
16	3183	0.12	1	3183
17	3183	0.12	2	−3183
18	3183	0.12	3	0
19	4775	0.1	1	4775
20	4775	0.1	2	−4775
21	4775	0.1	3	0
22	3979	0.1	1	3979
23	3979	0.1	2	−3979
24	3979	0.1	3	0
25	3183	0.1	1	3183
26	3183	0.1	2	−3183
27	3183	0.1	3	0

**Table 3:** Results of the statistical analysis, ANOVA, for the hole straightness and roundness errors.

	Straightness Error						Roundness Error					
Source	SS	DF	MS	F Value	*p* Value	PC	SS	DF	MS	F Value	*p* Value	PC
Model	1001.4231	9	111.2692	4.8885	0.0024	—	241.6086	9	26.8454	8.1059	0.0001	—
Constant	62.4926	1	62.4926	2.7456	0.1159	—	133.0856	1	133.0856	40.1850	0.0000	—
*n*	5.0843	1	5.0843	0.2234	0.6425	0.93	60.9210	1	60.9210	18.3950	0.0005	17.45
*n* ^2^	22.7492	1	22.7492	0.9995	0.3315	4.17	31.3793	1	31.3793	9.4749	0.0068	8.99
$f_{n}$	161.1526	1	161.1526	7.0801	0.0165	29.53	98.6792	1	98.6792	29.7960	0.0000	28.27
$f_{n}$ ^2^	229.4017	1	229.4017	10.0785	0.0055	42.03	63.8091	1	63.8091	19.2671	0.0004	18.28
Strategy	0.4898	1	0.4898	0.0215	0.8851	0.09	16.1376	1	16.1376	4.8727	0.0413	4.62
Strategy ^2^	102.2620	1	102.2620	4.4928	0.0491	18.74	15.8030	1	15.8030	4.7717	0.0432	4.53
$n\cdot f_{n}$	13.2300	1	13.2300	0.5812	0.4563	2.42	45.6300	1	45.6300	13.7779	0.0017	13.07
n·Strategy	2.8578	1	2.8578	0.1256	0.7274	0.52	10.0853	1	10.0853	3.0452	0.0990	2.89
$f_{n}\cdot \mathrm{Strategy}$	8.5773	1	8.5773	0.3768	0.5474	1.57	6.6034	1	6.6034	1.9939	0.1760	1.89
Error	386.9435	17	22.7614	—	—	27.87	56.3010	17	3.3118	—	—	18.90
Total	1388.3667	26	—	—	—	100.00	297.9096	26	—	—	—	100.00

**Table 4:** Results of the statistical analysis, ANOVA, for the hole cylindricity and diameter errors.

	Cylindricity Error						Diameter Error					
Source	SS	DF	MS	F Value	*p* Value	PC	SS	DF	MS	F Value	*p* Value	PC
Model	1197.8067	9	133.0896	7.0541	0.0003	—	2058.5104	9	228.7234	13.3858	0.0000	—
Constant	304.1953	1	304.1953	16.1231	0.0009	—	323.3141	1	323.3141	18.9216	0.0004	—
*n*	242.4367	1	242.4367	12.8497	0.0023	22.07	305.8570	1	305.8570	17.9000	0.0006	25.29
*n* ^2^	274.1171	1	274.1171	14.5289	0.0014	24.95	236.3222	1	236.3222	13.8305	0.0017	19.54
$f_{n}$	255.8686	1	255.8686	13.5617	0.0018	23.29	228.5186	1	228.5186	13.3738	0.0020	18.89
$f_{n}$ ^2^	265.3350	1	265.3350	14.0634	0.0016	24.15	201.0674	1	201.0674	11.7673	0.0032	16.62
Strategy	8.3119	1	8.3119	0.4406	0.5158	0.76	0.1563	1	0.1563	0.0091	0.9249	0.01
Strategy ^2^	14.7133	1	14.7133	0.7798	0.3895	1.34	120.7119	1	120.7119	7.0645	0.0166	9.98
$n\cdot f_{n}$	12.2008	1	12.2008	0.6467	0.4324	1.11	110.4133	1	110.4133	6.4618	0.0211	9.13
n·Strategy	1.5686	1	1.5686	0.0831	0.7766	0.14	0.8328	1	0.8328	0.0487	0.8279	0.07
$f_{n}\cdot \mathrm{Strategy}$	24.1237	1	24.1237	1.2786	0.2739	2.20	5.5642	1	5.5642	0.3256	0.5757	0.46
Error	320.7400	17	18.8671	—	—	21.12	290.4792	17	17.0870	—	—	12.37
Total	1518.5467	26	—	—	—	100.00	2348.9896	26	—	—	—	100.00

**Table 5:** Grey relational coefficients and Grey relational grade.

Experiment No.	*n*, rev/min	$f_{n},\;\mathrm{mm}/\mathrm{rev}$	Strategy No.	CYL	STR	RON	DE	Grade	Rank
1	4775	0.14	1	1.000	0.902	0.651	0.969	0.880	1
2	4775	0.14	2	0.965	1.000	0.532	1.000	0.874	2
3	4775	0.14	3	0.600	0.487	0.401	0.583	0.518	9
4	3979	0.14	1	0.455	0.531	0.354	0.473	0.453	17
5	3979	0.14	2	0.477	0.551	0.377	0.606	0.503	10
6	3979	0.14	3	0.650	0.812	0.345	0.587	0.598	3
7	3183	0.14	1	0.440	0.471	0.414	0.474	0.450	18
8	3183	0.14	2	0.521	0.563	0.378	0.501	0.491	13
9	3183	0.14	3	0.550	0.589	0.369	0.493	0.500	11
10	4775	0.12	1	0.587	0.553	0.494	0.450	0.521	8
11	4775	0.12	2	0.587	0.519	0.369	0.508	0.496	12
12	4775	0.12	3	0.457	0.465	0.401	0.540	0.466	14
13	3979	0.12	1	0.368	0.352	0.333	0.371	0.356	26
14	3979	0.12	2	0.402	0.512	0.388	0.410	0.428	21
15	3979	0.12	3	0.333	0.333	0.335	0.333	0.334	27
16	3183	0.12	1	0.452	0.411	0.403	0.391	0.414	23
17	3183	0.12	2	0.407	0.391	0.361	0.466	0.406	24
18	3183	0.12	3	0.419	0.438	0.333	0.369	0.390	25
19	4775	0.1	1	0.635	0.628	0.542	0.469	0.568	4
20	4775	0.1	2	0.509	0.583	0.542	0.492	0.532	7
21	4775	0.1	3	0.594	0.476	0.411	0.372	0.463	15
22	3979	0.1	1	0.433	0.485	0.476	0.359	0.438	20
23	3979	0.1	2	0.437	0.512	0.501	0.395	0.461	16
24	3979	0.1	3	0.441	0.395	0.479	0.350	0.416	22
25	3183	0.1	1	0.452	0.409	0.464	0.447	0.443	19
26	3183	0.1	2	0.395	0.374	1.000	0.398	0.542	6
27	3183	0.1	3	0.487	0.387	0.984	0.391	0.562	5

## Data Availability

Data sharing is not applicable.

## Nomenclature

n	spindle speed, rpm
*n_n_*	tool speed, rpm
$f_{n}$	feed per revolution, mm/rev
SS	sum of squares
DF	degrees of freedom
MS	mean square
CYL	cylindricity error, µm
STR	straightness error, µm
RON	roundness error, µm
DE	diameter error, µm
*p*	significance
PC	percentage contribution
LN_2_	liquid nitrogen
*f*	feed rate, mm/min
*v_c_*	cutting speed, m/min
VHM HPC	cemented carbide high performance
ANOVA	analysis of variance
HSS	high speed steel
MPE_E	length accuracy
MPE_P	probe ball accuracy
MPE_RON_t_	maximum permissible error of roundness
